# Phytochemistry, Pharmacology and Molecular Mechanisms of Herbal Bioactive Compounds for Sickness Behaviour

**DOI:** 10.3390/metabo12121215

**Published:** 2022-12-02

**Authors:** Ghallab Hamoud Sinhat Alotaibi, Thippeswamy Boreddy Shivanandappa, Maheswari Chinnadhurai, Sudharshan Reddy Dachani, Mahmad Dabeer Ahmad, Khalid Abdullah Aldaajanii

**Affiliations:** 1 Department of Pharmaceutical Sciences, College of Pharmacy, Shaqra University, Al-Dawadmi Campus, Al-Dawadmi 11961, Saudi Arabia; 2 Department of Biomedical Science, College of Pharmacy, Shaqra University, Al-Dawadmi Campus, Al-Dawadmi 11961, Saudi Arabia; 3 Department of Pharmacy Practice, College of Pharmacy, Shaqra University, Al-Dawadmi Campus, Al-Dawadmi 11961, Saudi Arabia

**Keywords:** sickness behaviour, phytomedicine, natural products, lipopolysaccharide, acute infections

## Abstract

The host’s response to acute infections or tissue injury is a sophisticated and coordinated adaptive modification called sickness behaviour. Many herbs have been studied for their ability to protect animals against experimentally induced sickness behaviour. However, there is a lack of knowledge and experimental evidence on the use of herbal bioactive compounds (HBACs) in the management of sick behaviour. The goal of this review is to provide a concise summary of the protective benefits and putative mechanisms of action of phytochemicals on the reduction of lipopolysaccharide (LPS)-induced sickness behaviour. Relevant studies were gathered from the search engines Scopus, ScienceDirect, PubMed, Google Scholar, and other scientific databases (between 2000 and to date). The keywords used for the search included “Lipopolysaccharide” OR “LPS” OR “Sickness behaviour” OR “Sickness” AND “Bioactive compounds” OR “Herbal medicine” OR “Herbal drug” OR “Natural products” OR “Isolated compounds”. A total of 41 published articles that represented data on the effect of HBACs in LPS-induced sickness behaviour were reviewed and summarised systemically. There were 33 studies that were conducted in mice and 8 studies in rats. A total of 34 HBACs have had their effects against LPS-induced changes in behaviour and biochemistry investigated. In this review, we examined 34 herbal bioactive components that have been tested in animal models to see if they can fight LPS-induced sickness behaviour. Future research should concentrate on the efficacy, safety, and dosage needed to protect against illness behaviour in humans, because there is a critical shortage of data in this area.

## 1. Introduction

Sickness behaviour is a complex and coordinated adaptive change initiated by the host to respond to acute infections or tissue injury [1,2,3,4]. Malaise, hyperalgesia, fever, lethargy, social withdrawal, inhibition, decreased locomotor activity, exploration, grooming, loss of libido, anhedonia, sleepiness, anorexia weight loss, disturbed concentration, and anxiety are part of the typical sickness behavioural pattern [3,5]. Even though neuronal receptors for bacteria and viruses do not exist, the presence of these microbes might elicit sick behaviour [6,7]. The immune system possesses receptors that can detect pathogens, which send a message to the brain via chemical massagers and cause altered behaviour in sick individuals [6,8].

Endocrine, autonomic, and behavioural alterations mediated by soluble proteins released at the site of infection or injury, such as proinflammatory cytokines, describe sickness behaviour [7,9]. Interleukin (IL)-1, IL-6, and tumour necrosis factor (TNF) are among the vital proinflammatory cytokines that activate immune cell (macrophages and dendritic cells) release [5,8]. Proinflammatory cytokines are crucial for controlling the immune system and coordinating cell-mediated immune responses [5]. To prevent and treat intracellular infections, proinflammatory cytokines affect immune cell growth, activation, differentiation, and homing to infection sites [8,9].

**Figure 1:**
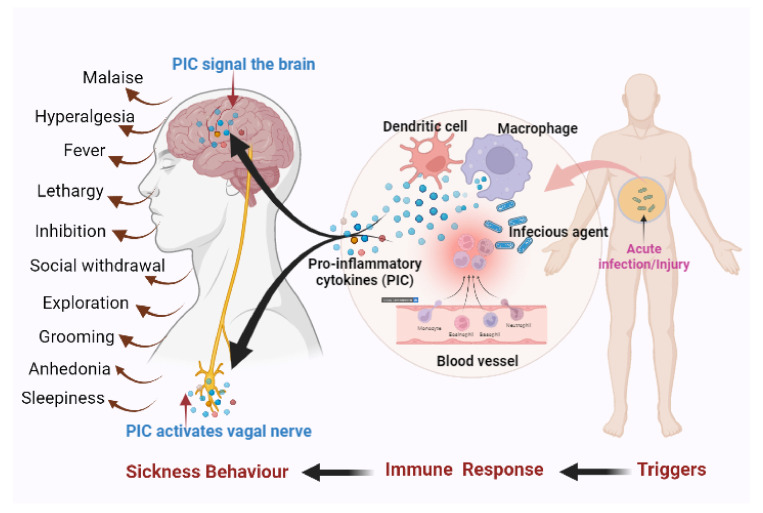
Role of the host immune system and proinflammatory cytokines in sickness behaviour. Acute infection or tissue injury acts as a trigger for the innate immune system. Dendritic cells and macrophages accumulate at the site of the infection or injury. The activated dendritic cells and macrophages release proinflammatory cytokines (PICs). The peripheral cytokines activate the vagal nerve, and some of the cytokines cross the blood–brain barrier and activate additional cytokine release from the brain. The brain is signalled by the released cytokines to start a series of behaviours (i.e., sickness behaviours). Figure created with the help of BioRender.com.

Proinflammatory cytokines not only coordinate peripheral inflammatory responses but also send signals to the brain that cause alteration in behaviour [7,9]. Cytokines activate afferent vagal nerves by binding with cells in the vagal paraganglia, and they can pass the blood–brain barrier (BBB), causing the central nervous system (CNS) to generate and release more proinflammatory cytokines [7,9,10]. The brain is signalled by the released cytokines to start a series of behaviours known as sickness behaviours [7,9]. A schematic representation of the role of the host immune system and proinflammatory cytokines in sickness behaviour is shown in Figure 1.

**Figure 2:**
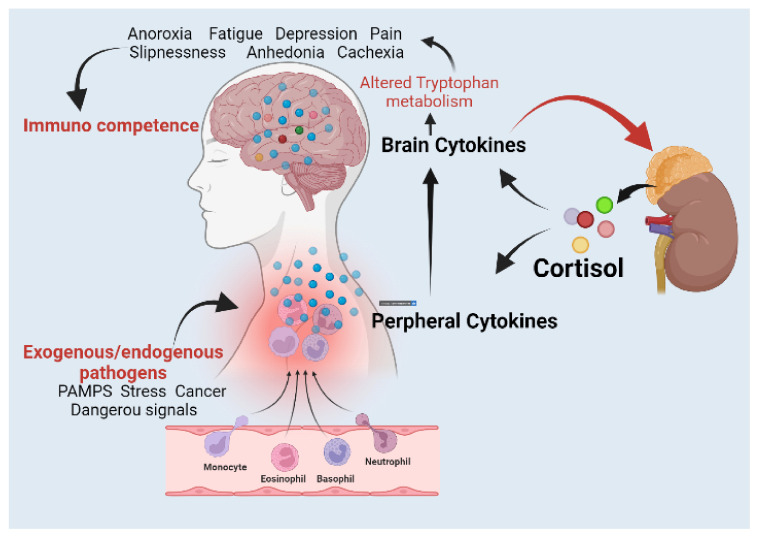
Bidirectional linkages between immunological events and psychoneuroendocrine states. The innate immune system can be activated by a variety of stimuli, including pathogen-associated molecular patterns (PAMPs), danger signals, and stress, increasing peripheral cytokines. The hypothalamic–pituitary–adrenal axis and neuronal circuits involved in the regulation of sleep, hunger, metabolism, emotion, and cognition are activated by brain cytokines. Cortisol inhibits the generation and activity of cytokines in both the peripheral and central nervous systems. Immunocompetence can be influenced by changes in the effect as well as other physiological systems, including metabolism and sleep, which influence the microbial load and, ultimately, the degree of activation of the innate immune system [9]. Figure created with the help of BioRender.com.

Centrally generated cytokines are believed to alter brain structures that control thermoregulation, metabolism, and behaviour via volume transmission [7,9]. As a result, the brain-based elements of the immune system are developed. The expression and actions of cytokines in the brain and other tissues are regulated by the adrenal cortex’s synthesis of glucocorticoids in response to the effects of cytokines on the hypothalamus [7]. Repetitive or chronic stimulation of the cytokine system can contribute to the development of mood disorders caused by cytokine-induced alterations in tryptophan metabolism [3,4,10]. Figure 2 depicts the bidirectional linkages between immunological events and psychoneuroendocrine states.

Sickness can be managed for a social animal by increasing sensitivity to dangerous social events and boosting approach-related behaviour toward close others who might be able to help [9]. The connection between the immune system and the CNS is a key aspect of host defence. Sickness behaviour affects the immune system and improves recovery [6]. Because inflammation is a powerful organiser of social behaviour, it has an impact on immune system management [6]. In reaction to various forms of social separation, the immune system upregulates proinflammatory response genes to prepare the body for more sensitive settings [6].

The above findings throw light on the mechanisms and methods for managing nonspecific symptoms of sickness, which can occur in a range of diseases linked to inflammation and lead to pathological mood and altered cognition [9]. Even though sickness behaviour is part of the immune reaction for the better healing of infection or injury, the sufferers feel discomfort if it persists for a longer time. Hence, sick behaviour should be treated to overcome the social, cognitive, and mental alterations in sufferers.

Many herbal medicines have been evaluated for their protective action against experimentally induced sickness behaviour in animals [1,11]. Nonetheless, there is a scarcity of knowledge and experimental data on the use of herbal bioactive compounds (HBACs) in sickness behaviour. The purpose of this review was to look at the present scientific literature on HBACs that protect experimental animals against sickness caused by lipopolysaccharide.

### Lipopolysaccharide (LPS)-Induced Sickness Behaviour Model

Several animal models are used for the preclinical evaluation of the effect of drugs on sickness behaviour. Among others, lipopolysaccharide (LPS)-induced sickness behaviour in rodents is most utilised in preclinical research. Rats and mice are widely utilised as experimental animals in the study of sickness behaviour.

**Figure 3:**
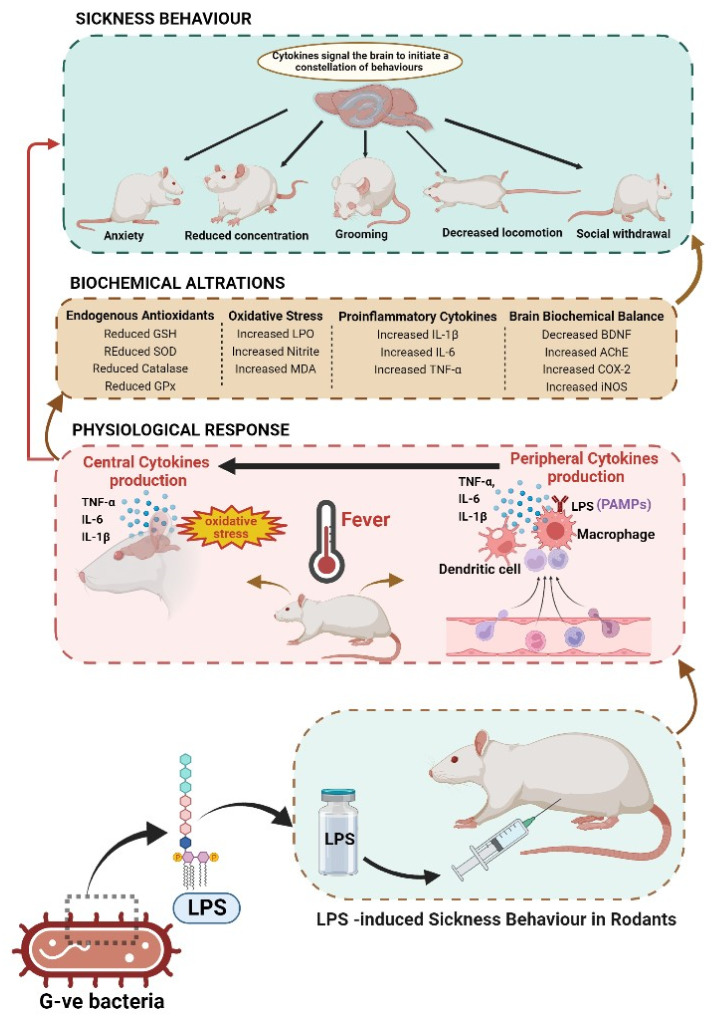
Physiological, behavioural, and biochemical alterations in LPS-induced sickness behaviour in rodents. Figure created with the help of BioRender.com.

LPS, a component of the cell walls of Gram-negative bacteria, is crucial for host–pathogen interactions with the innate immune system during infection [12]. Injection of LPS into rodents mimics the imperative aspects of Gram-negative bacterial infections, such as activating the Toll-like receptor 4 (TLR-4, pattern recognition receptor) [13]. Hence, LPS is often used to induce sickness behaviour in animals, which mimics sickness behaviour in humans [13,14,15]. By attaching to immune cells, LPS functions as a pathogen-associated molecule pattern (PAMP) [11] and activates nuclear factor κB (NFκB) to increase the expression of TNF-α, IL-6, and IL-1β [10,11]. In the CNS, microglia and macrophages generate cytokines and induce neuroinflammation and sickness behaviour [14,15]. In CNS, peroxides and reactive oxygen species (ROS) are produced in large numbers as a result of a rapid inflammatory response initiated by LPS [14,15]. When the levels of peroxides and ROS exceed the natural antioxidant defences, oxidative stress-mediated disease results [14,15]. In the brain, lipid peroxidation targets polyunsaturated fatty acids [10,11,16]. The detailed physiological, behavioural, and biochemical alterations in LPS-induced sickness behaviour in rodents are shown in Figure 3.

## 2. Methods

The relevant studies were gathered from the search engines Scopus, ScienceDirect, PubMed, Google Scholar, and other scientific databases (between 2000 and to date). The keywords used for the search included “Lipopolysaccharide” OR “LPS” or “Sickness behaviour” OR “Sickness” and “Bioactive compounds” OR “Herbal medicine” OR “Herbal drug” OR “Natural products” OR “Isolated compounds”.

Articles published only in the English language were considered, and conference abstracts and articles other than English were excluded. Studies conducted only on pure bioactive compounds were included in this review. Duplicate studies were deleted from the various databases. This review included a total of 41 published articles after applying the inclusion and exclusion criteria.

## 3. Results

### 3.1. Selection of Articles

**Figure 4:**
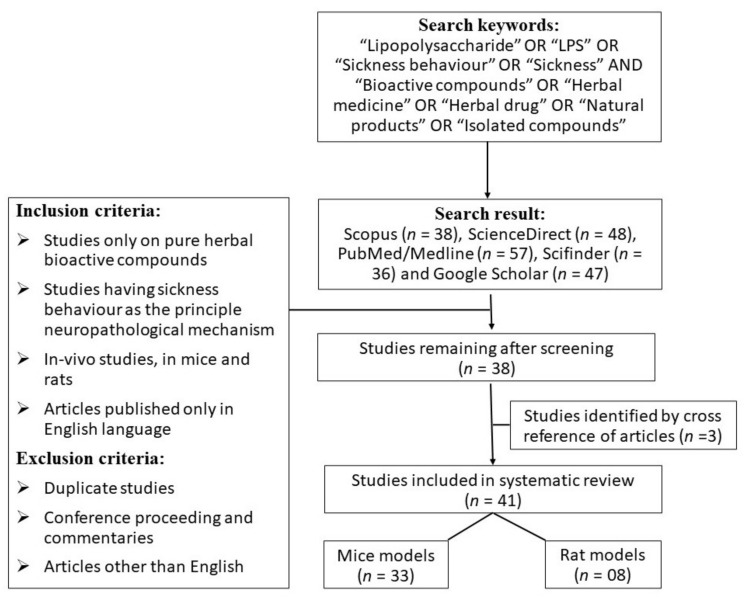
Search strategy.

The electronic searches resulted in the following number (*n*) of articles in the databases: Scopus (*n* = 38), ScienceDirect (*n* = 48), PubMed/Medline (*n* = 57), Scifinder (*n* = 36), and Google Scholar (*n* = 47). This study also considered three articles that were supported by cross-references from other studies. Based on the inclusion and exclusion criteria, 41 articles out of 229 were chosen for this review. Most of the studies administered LPS through the i.p. route; in a few studies, LPS was directly injected into the brain and was conducted in mice (*n* = 33) or rat (*n* = 8) models. The search approach and descriptions of the papers containing HBACs are compiled in Figure 4 and Table 1, respectively.

**Figure 5:**
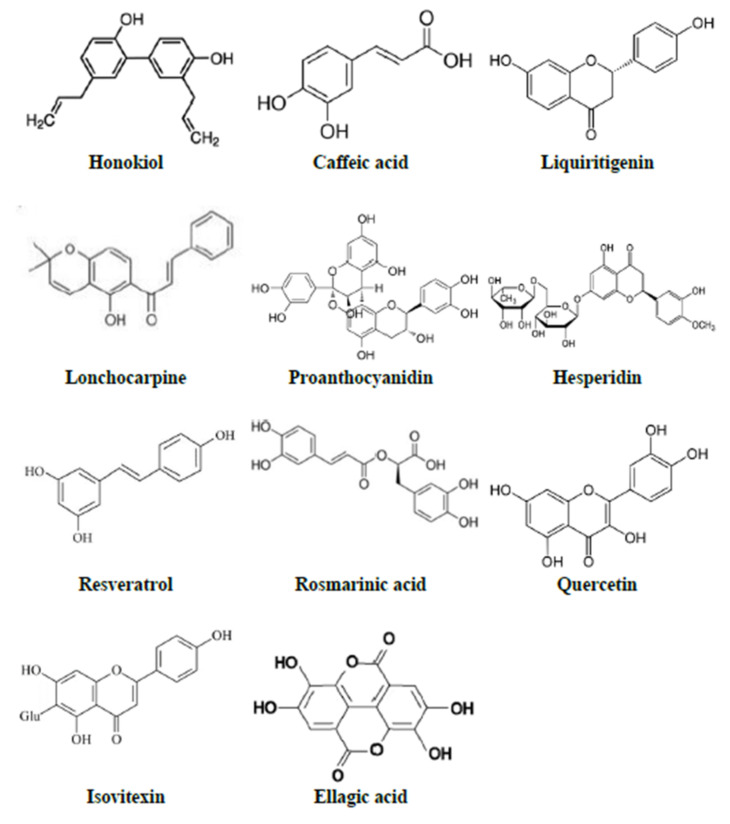
Phenolic or polyphenolic compounds effective against LPS-induced sickness behaviour in rodents.

**Figure 6:**
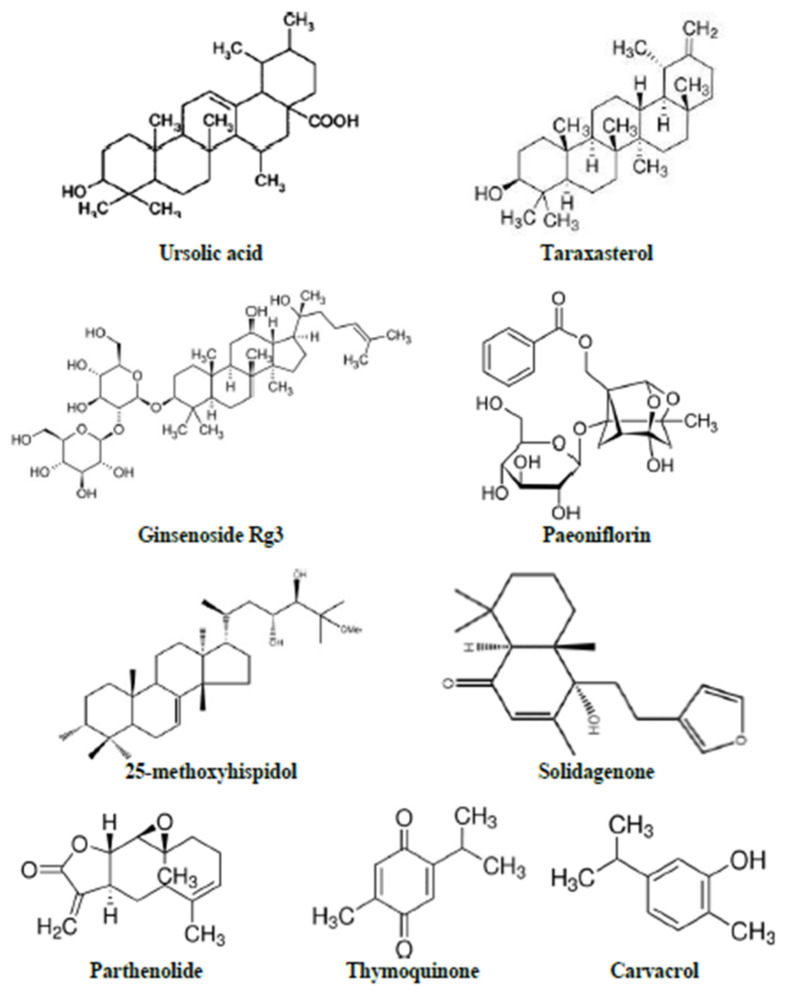
Terpenes or terpenoids compounds effective against LPS-induced sickness behaviour in rodents.

A total of 34 HBACs have been evaluated for their effects against LPS-induced behavioural and biochemical alterations. The HBACs include phenolic or polyphenolic compounds (honokiol, caffeic acid, liquiritigenin, lonchocarpine, proanthocyanidin, hesperidin, resveratrol, rosmarinic acid, quercetin, isovitexin, and ellagic acid; Figure 5), terpenes or terpenoids (ursolic acid, taraxasterol, ginsenoside Rg3, 25-methoxyhispidol, solidagenone, paeoniflorin, parthenolide, thymoquinone, and carvacrol; Figure 6), lignan (macranthol and gomisin N), curcumin, mangiferin, esculetin, embelin, paeonol, trans-astaxanthin, 2,3,4′,5-tetrahydroxystilbene-2-O-D-glucoside, methyl jasmonate, gentiopicroside, selanylimidazopyridine, diallyl disulfide, and gypenosides.

### 3.2. HBACs Conferring Protection in Mice

#### 3.2.1. Ursolic Acid

Ursolic acid is a pentacyclic triterpenoid that can be found in the leaves, flowers, berries, and fruits of many medicinal plants, including apples, bilberries, cranberries, elder flower, peppermint, lavender, oregano, thyme, hawthorn, and prunes [54]. Ursolic acid has antioxidant, anti-inflammatory, antibacterial, and antifungal properties. Wang and colleagues looked at how ursolic acid affected LPS-induced cognitive impairments in mice [47]. Ursolic acid protects animals from cognitive deficits induced by LPS. Ursolic acid protects mice by inhibiting p38/NF-B-driven inflammatory pathways in the brain [47].

#### 3.2.2. Taraxasterol

Taraxasterol (anthesterin) is pentacyclic-triterpene obtained from the plant *Taraxacum officinale* (Family: Asteraceae) [55]. The reported pharmacological actions of taraxasterol include anti-inflammatory, antioxidative, and anticarcinogenic properties. Taraxasterol was evaluated by Zhang et al. for its effect against LPS-induced endotoxic shock [51]. Taraxasterol protects mice from endotoxic shock by modulating inflammatory responses [51]. The administration of taraxasterol attenuated the altered levels of IL-1β, IL-6, TNF-α, IFN-g, and PGE2 in LPA-treated animals [51].

#### 3.2.3. Curcumin

Curcumin (diferuloylmethane) is the main active constituent of *Curcuma longa* (Family: Zingiberaceae) [56]. Wang et al. evaluated the antidepressant activity of curcumin in LPS-treated mice. Treatment with curcumin attenuates iNOS, cytokines, and the expression of COX-2 mRNA via the NF-κB signalling pathway and protects animals from LPS-induced depressive-like behaviour [48]. In addition, Sorrenti et al. evaluated curcumin in acute neuroinflammation and long-term memory impairment in LPS-treated mice [42]. According to the authors, curcumin protects rats from LPS-induced memory loss and acute neuroinflammation [42]. Piperine, a main alkaloidal of *Piper nigrum*, inhibits glucuronidation and improves the bioavailability of curcumin [57,58]. Jangra et al. reported that piperine enhances the efficacy of curcumin in protecting neurobehavioral and neurochemical impairments in LPS-treated mice [26]. Piperine increases curcumin bioavailability, which improves its biological performance in LPS-treated mice [26].

#### 3.2.4. Honokiol

Honokiol is a polyphenolic compound that can be obtained from *Magnolia grandiflora* (Family: Magnoliaceae) [59]. Honokiol offers antianxiety, antipain, and anti-epileptic properties [59]. Sulakhiya et al. reported the abrogative effect of honokiol in depressives-like behaviour in LPS-treated rats by reducing neuroinflammation and oxido-nitrosative stress in mice [44]. In addition, as per the study conducted by Sulakhiya et al., honokiol offers beneficial effects on anxiety and liver damage in LPS-treated mice [24]. In mice, Honokiol had a protective effect against anxiety-like behaviour and liver damage caused by LPS. Honokiol inhibits cytokine generation, oxidative stress, and the loss of brain-derived neurotrophic factor (BDNF) [24].

#### 3.2.5. Mangiferin

Mangiferin is C-glucosylxanthone found in the root, bark, and leaves of *Mangifera indica* (Family: Anacardiaceae) [60]. Mangiferin has antioxidant, antibacterial, antiallergic, immunomodulatory, anticancer, antidiabetic, and hypocholesterolemic actions [60]. Jangra and colleagues investigated the influence of mangiferin on depressive- and anxiety-like behaviour in LPS-treated mice [25]. Mangiferin protects mice from depressed and anxiety-like behaviour by inhibiting neuroinflammation, oxidative stress, and preventing BDNF depletion in the brain [25].

#### 3.2.6. Esculetin

Esculetin is a coumarin derivative found in *Artemisia scoparia*, *Artemisia capillaries*, *Ceratostiggma willmottianum*, and *Citrus limonia* [61]. Esculetin is well known for its pleiotropic biological activity, which includes antioxidant, inhibition of xanthine oxidase, platelet aggregation, and anticancer activities [61]. Sulakhiya et al. evaluated the antianxiety and antidepressant action of esculetin in LPS-treated mice [62]. Esculetin alleviated LPS-induced anxiety and sadness in rats by reducing neuroinflammation, oxidative stress, and plasma cortisol levels [62]. Esculetin reduced LPS-induced neuroinflammatory processes and depressive-like behaviour in mice [53]. According to the author, the impact of esculetin may be attributed to the suppression of the NF-B pathway and the stimulation of BDNF/TrkB signalling [53].

#### 3.2.7. Caffeic Acid

Caffeic acid is a polyphenolic compound found in a wide range of plants and foods, such as coffee, wine, and tea [63]. Caffeic acid has antioxidant, anti-inflammatory, and anticarcinogenic properties [63]. Mallik et al. evaluated caffeic acid on sickness behaviour in LPS-treated mice [37]. Caffeic acid (30 mg/kg) protected from LPS-induced sickness behaviour and neuroinflammation in mice [37]. Caffeic acid reduced peripheral and central cytokine levels as well as the oxidative stress caused by LPS [37].

#### 3.2.8. Embelin

Embelin is alkyl-substituted hydroxyl benzoquinone found in *Embelia ribes* Burm [64]. Embelin possesses neuroprotective effects against experimentally induced neurotoxicity in animals [64,65]. Shaikh et al. reported the beneficial effect of embelin in sickness behaviour in LPS-treated mice [11]. The authors reported that the antioxidant properties of embelin are responsible for its protective action against LPS-induced sickness behaviour [11].

#### 3.2.9. Gomisin N

Gomisin N is a lignan extracted from *Schisandra chinensis* (Family: Schisandraceae) Baill’s dried fruits [66]. *Schisandra chinensis* has long been used in traditional Chinese and Kampo medicine for liver disorders. Gomisin N has antioxidant, anti-inflammatory, and hepatoprotective effects in vivo and in vitro. Gomisin N reduces depressive-like behaviour and interest loss caused by LPS. Gomisin N’s anti-inflammatory and antineuronal actions are most likely due to the reduction in neural activation and inflammation in the PVN and CeA [18].

#### 3.2.10. Liquiritigenin

Liquiritigenin is a flavanone identified from *Glycyrrhiza uralensis* and found in many plants, including *Glycyrrhiza glabra* [67]. Su et al. discovered that liquiritigenin protects mice from depressive-like behaviour caused by LPS [43]. Liquiritigenin’s anti-inflammatory properties and impact on the BDNF/TrkB signalling pathway are thought to be the cause of its antidepressant effects [43].

#### 3.2.11. Paeonol

Paeonol, the active ingredient of the moutan cortex, has been widely researched as an antioxidant, anti-inflammatory, antidiabetic, antiatherosclerosis, and antimutagenic agent [68]. Paeonol reduces depressive-like behaviour in mice treated with LPS [45]. It was observed that paeonol could successfully reverse changes in the levels of TNF-α, IL-6, 5-HT, and NE. Paeonol also inhibited the expression of tropomyosin-related kinase B (TrkB), nuclear factor-κB (NF-κB), and BDNF in the hippocampus [45].

#### 3.2.12. Trans-Astaxanthin

Algae, plants, a few fungi, and bacteria all contain large amounts of the red carotenoid pigment trans-astaxanthin [28]. Trans-astaxanthin has been shown to have neuroprotective properties in a variety of neurodegenerative illnesses [28]. Trans-astaxanthin has been shown in animal studies to reduce LPS-induced neuroinflammation and depressive-like behaviour [69]. Trans-astaxanthin inhibited iNOS, nNOS, and COX-2 expression as well as NO levels in the hippocampus and prefrontal cortex by regulating NF-κB [69]. Furthermore, it has been observed that trans-astaxanthin has an antidepressant-like impact on the serotonergic system [28].

#### 3.2.13. 2,3,4′,5-Tetrahydroxystilbene-2-O-β-D-glucoside

The major active ingredient in *Polygonum multiflorum* Thunb is 2,3,4′,5-tetrahydroxystilbene-2-O-D-glucoside (TSG) [70]. TSG has been demonstrated to have hypotensive, anti-ageing, anti-inflammatory, hypolipidemic, cardioprotective, and neuroprotective actions [70]. Chen et al. reported the preventive action of TGS against LPS-induced depressive behaviours in mice [20]. TSG pretreatment at 30 and 60 mg/kg reduced IL-1β, IL-6, TNF-α, and oxido-nitrosative stress production in the hippocampus and prefrontal cortex [20].

#### 3.2.14. Ginsenoside Rg3

Ginsenoside Rg3 is a tetracyclic triterpenoid and a glycoside found in *Panax ginseng* (red ginseng, Family: Araliaceae), and it has antioxidant, anti-inflammatory, and immunomodulatory properties [71]. Kang and colleagues found that ginsenoside Rg3 suppressed depression-like behaviour and neuroinflammation produced by LPS in mice [30]. The protective effect was achieved by inhibiting neuroinflammatory disturbances and regulating TRP-KYN metabolism in both the brain and the peripheral nervous system [30].

#### 3.2.15. Lonchocarpine

Lonchocarpine is a phenylpropanoid derived from the plant *Abrus precatorius* (Family: Fabaceae) [72]. Lonchocarpine has antibacterial, anti-inflammatory, antiproliferative, and antiepileptic properties [72]. Jeong et al. investigated the role of lonchocarpine in LPS-induced neuroinflammation in mice [27]. The author reported that the anti-inflammatory action of lonchocarpine is attributed to its beneficial actions [27].

#### 3.2.16. Methyl Jasmonate

Methyl jasmonate is a hormone initially isolated from *Jasmonium grandiflorum* (Family: Oleaceae) essential oil [73]. Methyl jasmonate is known to have anti-amnesic, antinociceptive, adaptogenic, and antidepressant properties [17]. Adebesin et al. reported the antidepressant effect of methyl jasmonate in LPS-treated mice [17]. The authors reported that the observed effect of methyl jasmonate was attributed to the suppression of oxidative stress and TNF-α release [17].

#### 3.2.17. Proanthocyanidin

Proanthocyanidin is a phenolic chemical present in plant seeds, nuts, flowers, fruits, and bark [74,75]. Proanthocyanidin has been shown to have antioxidant, anti-inflammatory, anti-allergic, antiviral, antibacterial, anticarcinogenic, and vasodilatory properties [74,75]. Jiang et al. evaluated the efficacy of proanthocyanidin to modify depressed and anxiety-like behaviours in LPS-treated mice [17]. Proanthocyanidin reduced LPS-induced COX-2 and iNOS overexpression in the different regions of the brain by modulating NF-κB [17].

#### 3.2.18. Gentiopicroside

Gentiopicroside is an iridoid glucoside and one of the primary compounds enriched in *Gentiana Macrophylla* Pall roots (Family: Gentianaceae) [15]. Gentiopicroside has been shown to possess analgesic, anti-inflammatory, anticancer, lipid regulating, and antidepressant properties. Deng et al. reported the gentiopicroside abrogates depressive-like behaviour in mice induced by LPS [21]. The abrogative effect of gentiopicroside mediates through the tryptophan-degrading pathway [21].

#### 3.2.19. Selanylimidazopyridine

Selanylimidazopyridine has received a lot of interest lately because of its antioxidant properties and potential to guard against depression-like behaviours [76]. According to Domingues et al., selanylimidazopyridine targets neurotrophins and inflammatory/oxidative mediators to prevent LPS-induced depressive-like behaviour in mice [22].

#### 3.2.20. 25-methoxyhispidol

25-methoxyhispidol is a triterpenoid isolated from *Poncirus trifoliate* (Family: Rutaceae) immature fruit [77]. 25-Methoxyhispidol has anti-inflammatory, neuroprotective, and anticancer properties [41,77]. Shal et al. evaluated 25-methoxyhispidol for anxiety and depression in LPS-treated mice [42]. By lowering the levels of IL-1β, IL-6, and TNF-α in the brain, 25-methoxyhispidol reduced neuroinflammation [42]. Pretreatment with 25-methoxyhispidol reduced cortisol levels and avoided alterations in the granular layer thickness in the dentate gyrus [42].

#### 3.2.21. Macranthol

Macranthol is a triphenyl lignan derived from the plant *Illicium dunnianum* (Family: Schisandraceae) [78]. Macranthol has been reported to possess antidepressant action in a preclinical study [79]. Weng et al. reported the attenuating action of macranthol in depressive-like behaviours in LPS-treated mice [50]. The antidepressant action of macranthol is mediated by inhibiting neuroinflammation in the prefrontal cortex [50]. According to another study, macranthol stimulates hippocampal neuronal development in mice via the BDNF-TrkB-PI3K/Akt signalling pathway [80].

#### 3.2.22. Hesperidin

Hesperidin is a bioflavonoid found primarily in citrus fruit, such as lemon, grapefruit, orange, and tangerine [81]. Hesperidin has several pharmacological properties, including antihyperlipidemic, cardioprotective, antihypertensive, and antidiabetic effects. [81]. According to a study conducted by Kwatra et al., hesperidin was found to be protective against LPS-induced hippocampus and frontal brain damage in mice [32]. The authors reported that the TLR4/NF-κB, p38 MAPK/JNK, and Nrf2/ARE signalling pathways play important roles in the activity of hesperidin [32].

#### 3.2.23. Resveratrol

Resveratrol is a polyphenolic, non-flavonoid found in plants, such as rhubarb, grapes, mulberries, and peanuts [82]. Resveratrol provides numerous health benefits, including antioxidant, anti-inflammatory, antiplatelet, blood glucose-lowering, and anticancer effects [83]. A group of researchers from China reported that resveratrol reduces anxiety-like behaviour in LPS-treated mice [46]. The antianxiety effect of resveratrol is attributed to its attenuating effect on YAP-mediated neuro-inflammation and promoting hippocampal autophagy [46].

#### 3.2.24. Solidagenone

Solidagenone is a diterpenoid compound found in *Solidago chilensis* (Family: Asteraceae) that is used in folk medicine to treat pain and inflammatory diseases [84]. The aerial parts of *Solidago chilensis* are frequently used to treat burns and for their diuretic, analgesic, anti-inflammatory, antirheumatic, and healing properties. Solidagenone has anti-inflammatory, antigastroprotective, and immunomodulatory properties [85]. According to Locateli et al., solidagenone has antidepressant-like effects in LPS-treated mice [36]. The impact of solidagenone has been linked to the control of antioxidant systems and a decrease in the inflammatory process [36].

#### 3.2.25. Diallyl Disulfide

Diallyl disulfide is an organosulfur compound derived from *Allium sativum* (Garlic, family: Allium) [86]. Wei et al. reported that diallyl disulfide attenuates depression-like behaviour in mice treated with LPS [49]. The observed effect was attributed to its regulating effect on neuroinflammation and oxido-nitrosative stress [49]. Lu and colleagues also reported the beneficial effect of diallyl disulfide in LPS-induced depression in mice [87].

#### 3.2.26. Rosmarinic Acid

Rosmarinic acid is a polyphenol constituent identified in *Rosmarinus officinalis* (Family: Lamiaceae) and many culinary herbs [88]. Rosmarinic acid is an ester of caffeic acid and 2-hydroxy-dihydrocaffeic alcohol with antioxidant and anti-inflammatory properties. Thingore et al. reported the ameliorative effect of rosmarinic acid on oxidative stress and neuroinflammation in LPS-induced memory-impaired mice [38]. The increased levels of proinflammatory cytokines and apoptotic proteins were revived after pretreatment with rosmarinic acid [38].

### 3.3. HBACs Conferring Protection in Rats

#### 3.3.1. Paeoniflorin

One of the most important bioactive components of paeony (*Paeonia lactiflora*, Family: Paeoniaceae) is paeoniflorin, a monoterpene glucoside [89]. Kim and Ha evaluated paeoniflorin against LPS-induced oxidative stress and lipid metabolism in rats [31]. Administration of paeoniflorin regulated the levels of lipid profile (triglyceride, total lipid, total-cholesterol, and HDL-cholesterol) levels and protected animals from oxidative stress [31]. This study demonstrates that paeoniflorin markedly ameliorated LPS-induced oxidative stress and lipid metabolism in rats [31]. Significant body weight loss is part of the typical sickness behavioural pattern [3,5]. The loss of appetite and altered lipid and protein metabolism leads to a significant body weight loss during sickness behaviour [90].

#### 3.3.2. Parthenolide

Parthenolide is a sesquiterpene lactone found in the herb feverfew (*Tanacetum parthenium*, Family: Asteraceae) [91]. A group of German researchers evaluated the effect of Parthenolide (1 mg/kg) on fever, circulating cytokines, and markers of brain inflammation in LPS-treated rats [39]. Parthenolide reduced LPS-induced fever in rats, and the authors propose that inhibition of the peripheral circulating IL-6 and TNF-α, as well as direct central action on brain cells via partial inhibition of oxidative stress, the NFκB and NF-IL6 signalling pathways, and inhibition of cytokines at the brain was attributed to its action [40].

#### 3.3.3. Quercetin

Quercetin is a bioflavonoid present in a variety of plants and foods, including onions, apples, berries, green tea, and red wine, and is known to have powerful ROS-scavenging properties [40]. Three important functions of quercetin are antioxidant, anti-inflammatory, and immunomodulatory. Sah et al. studied quercetin for them LPS-induced-sickness behaviour in rats [40]. The authors conclude that administration of quercetin (2 and 25 mg/kg) significantly attenuates sickness behaviour induced by LPS by inhibiting oxidative stress and modulating cytokines production [40]. The effect of quercetin on LPS-induced abnormality was also evaluated in mice as an animal model. Liao and Lin administered quercetin intraperitoneally (0.06 μmol/mouse) to LPS-challenged mice [34]. Quercetin treatment protected mice from LPS-induced systemic inflammation [34].

#### 3.3.4. Thymoquinone

Thymoquinone is a monoterpene molecule found in the seeds of *Nigella sativa* L. (black cumin, family: Ranunculaceae) [92,93]. Antioxidant, anti-inflammatory, antihistaminic, antidiabetic, anticonvulsant, antimicrobial, and anticancer properties have been documented for thymoquinone [92,93]. Bargi and colleagues investigated the effect of thymoquinone in LPS-treated rats [19]. Thymoquinone reduced cytokine levels, oxidative stress status, and memory impairments caused by LPS in rats [19].

#### 3.3.5. Gypenosides

Gypenosides is saponin derived from *Gynostemma pentaphyllum* (*Jiaogulan,* Family: Cucurbitaceae) [94]. Gypenosides have been demonstrated to have anxiolytic and neuroprotective benefits in the treatment of depressive disorders [95]. Lee and a friend showed that in rats, gypenosides reduce lipopolysaccharide-induced neuroinflammation and memory loss [96]. Due to the fact of their anti-inflammatory actions and adequate modulation of NFκB/iNOS/TLR4/BDNF, gypenosides have been shown to have anxiolytic and neuroprotective benefits through increasing memory functions [96].

#### 3.3.6. Isovitexin

Isovitexin is the flavonoid abundantly found in the leaf of *Celtis sinensis* (Family: Cannabaceae). Isovitexin is known for its antineoplastic, antioxidant, anti-inflammatory, and neuroprotective effects [97]. Liu et al. reported isovitexin beneficial action on neuroinflammation induced by LPS [35]. The authors reported that isovitexin regulates microglial polarisation in LPS-induced neuroinflammation via activating the CaMKKβ/AMPK-PGC-1α signalling axis [35].

#### 3.3.7. Carvacrol

Carvacrol is a phenolic monoterpenoid found in the essential oils of Lamiaceae plant species such as *Citrus aurantium bergamia, Thymus vulgaris, Origanum vulgare, Lepidium flavum,* etc., [98]. Anticancer, antifungal, antibacterial, antioxidant, anti-inflammatory, vasorelaxant, hepatoprotective, and spasmolytic properties have been found for carvacrol [99]. Carvacrol inhibits memory impairment and inflammation in LPS-treated rats, the carvacrol showed anti-inflammatory effects mediated by BDNF and TLR4 regulation [33].

#### 3.3.8. Ellagic Acid

Ellagic acid is a polyphenolic substance obtained from nuts, raspberries, strawberries, wolfberries, blackberries, pomegranates, cranberries, pecans, and other plant foods [100]. Ellagic acid is reported to possess antioxidant, antimutagenic, hepatoprotective, and anticancer properties [100]. Dornelles and coworkers reported the attenuating activities of ellagic acid in cognitive impairment and neuroinflammation in LPS-treated rats [23]. Ellagic acid reduced glial cell expression, phosphorylated Tau, oxidative damage, and acetylcholinesterase activity [23].

## 4. Mechanism of Action(s) of HBACs against LPS-Induced Sickness Behaviour

**Figure 7:**
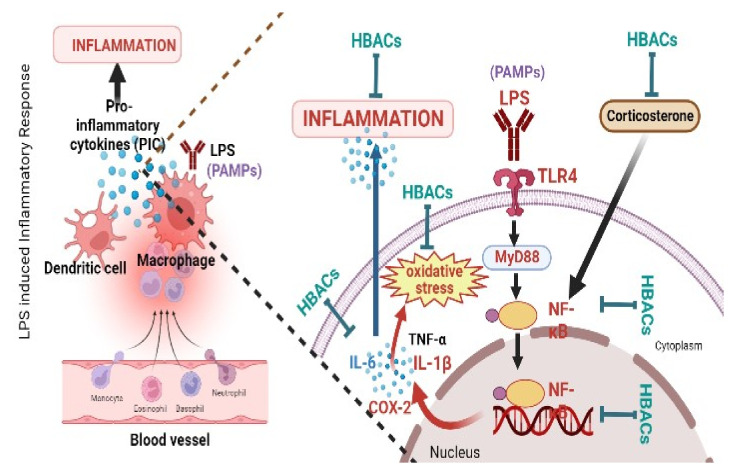
Molecular mechanism of action(s) of HBACs against LPS-induced sickness behaviour in rodents. PAMPs: pathogen-associated molecular patterns, TLR4: Toll-like receptor 4; IL-1β: interleukin-1β; IL-6: interleukin-6; TNF-α: tumour necrosis factor-α; and COX-2: cyclooxygenase-2. Figure created with the help of BioRender.com.

LPS is a pathogen-associated molecular pattern (PAMP) that allows bacteria to be identified by pattern recognition receptors on certain host receptors (PRRs). LPS works as a toxin by activating the Toll-like receptors (TLRs) signalling pathway, which promotes pathogenic inflammatory responses by increasing the nuclear translocation of NF-B and triggering the production of proinflammatory cytokines, such as IL-1β, IL-6, and TNF-α. High LPS concentrations induce the production of proinflammatory mediators, which can result in oxidative stress. It is thought that ROS are involved in the mechanism of LPS toxicity. The majority of HBACs reduced the oxidative and nitrative stress and attenuated proinflammatory cytokines IL-1β, IL-6, and TNF-α, as a result, suppressing the neuronal inflammation in the LPS-treated animals. Corticoids produced in response to the hypothalamic effects of proinflammatory cytokines control cytokine expression and function [7]. Some of the HBACs (esculetin and methyl jasmonate) suppress the corticosterone and reduce the further release of proinflammatory cytokines. In addition, some HBACs (ursolic acid, curcumin and proanthocyanidin) also inhibited the COX-2 enzyme in the brain. COX-2 is thought to be involved in the inflammatory response in the neurons inhibiting it and suppresses the inflammation. BDNF is a pleiotropic protein that modulates neurotransmitters and plays a role in memory and learning [101]. BDNF is necessary for the appropriate development of various nervous system components [102]. LPS administration reduces BDNF levels in the brain and affects memory and learning in animals [101]. Some of the HBACs (mangiferin, honokiol, liquiritigenin, paeonol, gypenosides, selanylimidazopyridine, carvacrol, and hesperidin) restored BDNF levels in LPS-treated animals and protected the animals from LPS-induced abnormalities. Certain HBACs showed their actions with multiple targets and protected the animals from LPS-induced toxicities. Table 2 and Figure 7 represent the molecular mechanism of action(s) of HBACs against LPS-induced sickness behaviour in rodents.

## 5. Conclusions and Future Perspectives

In this review, we looked at 34 herbal bioactive components that have been studied for their ability to combat LPS-induced illness in animal models. The majority of the researched herbal bioactive compounds induced a reduction in sickness behaviour signs in experimental animals, according to our review. Nonetheless, because most studies focused solely on its effects on sickness behaviour, the toxicological profiles of the herbal bioactive components are unknown. Furthermore, there is a severe dearth of data on the efficacy, safety, and required dosage to protect from sickness behaviour in humans, which should be the focus of future research. The potentials of herbal bioactive compounds should be studied for the development of novel medications as adjuvants or as a new armamentarium to augment sickness behaviour treatment.

## Figures and Tables

**Table 1:** Herbal bioactive compounds conferring protection against LPS-induced sickness behaviour in rodents.

Phytoconstituent	Isolated from	Reported Activities	Animal/Dose	Dose of LPS	Parameters Evaluated	Reference
Methyl Jasmonate	*Jasmonium grandiflorum*	Antinociceptive, anti-amnesic, and adaptogenic properties.	Male Swiss mice/5, 10, 20 mg/kg/7 days	830 mg/kg (i.p.)	Behavioural Sucrose preference test Tail suspension test and Forced swim test Locomotor activity Biochemical Estimation Corticosterone Glutathione Malondialdehyde Super oxide dismutase TNF-α	[17]
Gomisin N	*Schisandra chinensis* (Turcz.)	Antioxidant and protective effects against tissue injury of heart, liver, kidney, and brain.	Mice	500 mg/kg (i.p.)	Behavioural Object exploration test Forced swim test Locomotor activity Biochemical Estimation Griess assay c-Fos immunohistochemistry Quantitative real-time PCR MTS assay	[18]
Thymoquinone (Found in seeds of *Nigella sativa* L)	Purchased from Sigma-Aldrich	Anti-inflammatory and neuroprotective effects.	Male Wistar rats/2, 5, and 10 mg/kg (i.p.)	1 mg/kg/day (i.p.) for two weeks	Behavioural Morris water maze test Passive avoidance Biochemical Estimation IL-6 TNF-α MDA Thiol Superoxide dismutase Catalase Nitric oxide	[19]
2,3,5,4′-Tetrahydroxystilbene-2-O-β-D-glucoside (TSG) (Found in *Polygonum multiflorum* Thunb.)	Purchased from the National Institute for the Control of Pharmaceutical and Biological Products (Beijing, China)	Antioxidative, free radical scavenging, and antiplatelet activities.	Male ICR mice, 30 and 60 mg/kg (i.p.)	0.83 mg/kg (i.p.)	Behavioural Tail suspension test Forced swim test Open field test Biochemical Estimation IL-1β IL-6 TNF-α MDA GSH level BDNF Nitrite level	[20]
Gentiopicroside (Gent) (Found in *Gentiana rigescens*)	Purchased from Spring & Autumn Biologic Engineering Co., Ltd. (Nanjing, China)	Anti-inflammatory activity.	8–10 week old male BALB/C mice, 50 mg/kg (i.p.) once a day	0.5 mg/kg (i.p.)	Behavioural Forced swimming test Tail suspension test Open field test Biochemical Estimation IL-1β TNF-α Protein expression of NMDA receptors (Western blot)	[21]
3-((4-methoxyphenyl) selanyl)-2 phenylimidazo [1,2-a] pyridine (Selanylimidazopyridin)	Synthesised by the Laboratory of Clean Organic Synthesis (LASOL-UFPel)	Anti-inflammatory, antioxidant, antidepressant, antineuroinflammatory, and antioxidant.	Male Swiss mice, (MPI; 20 and 50 mg/kg, intragastrically)	0.83 mg/kg (i.p.)	Behavioural Open field test Forced swimming test Biochemical Estimation Lipid peroxidation Reactive oxygen species (ROS) BDNF TBARS level Gene expression	[22]
Ellagic acid (Found in strawberries, raspberries, blackberries, cherries, and walnuts)	Purchased from Sigma-Aldrich	Antioxidant, anti-Alzheimer’s, and anti-Parkinson’s activities.	Male Wistar rats, 100 mg/kg intragastric gavage	250 μg/kg (i.p.)	Behavioural Open field test Object recognition test Biochemical Estimation Lipid peroxidation Reactive oxygen species (ROS) Protein carbonylation T-SHs level GSH level Acetylcholinesterase activity TBARS measurement Protein carbonyl level	[23]
Esculetin (Found in *Artemisia eriopoda, Euphorbia decipiens*)	Purchased from Sigma-Aldrich	Antioxidant, anti-inflammatory, antiproliferative, and antidepressant and cognitive enhancer.	Mice 25 and 50 mg/kg (p.o.)	0.83 mg/kg (i.p.)	Behavioural Elevated plus maze Open field test Forced swim test Tail suspension test Biochemical Estimation Cytokines MDA level GSH level CORT IL-1β IL-6 TNF-α Oxidative stress	[24]
Mangiferin (Found in *Mangifera indica*)	Purchased from Sigma-Aldrich	Antioxidant, anti-inflammatory, and immunomodulatory activities.	Mice, 20 and 40 mg/kg (p.o.)	0.83 mg/kg (i.p.)	Behavioural Elevated plus maze Light–dark box Open field test Sucrose preference Biochemical Estimation IL-1β SOD Catalase MDA level GSH level Nitrite assay TNF-α BDNF	[25]
Curcumin and piperine	Purchased from Sigma-Aldrich	Antioxidant, anti-inflammatory, hepato- and nephroprotective activity, and antimicrobial and neuroprotective properties.	Male Swiss albino mice; curcumin alone—100, 200, and 400 mg/kg (p.o.); curcumin and piperine—20 mg/kg (p.o.)	0.83 mg/kg (i.p.)	Behavioural Elevated plus Maze Light–dark box test Open field test Sucrose preference test Tail suspension test Forced swimming test Biochemical Estimation IL-1β TBARS level CORT level MDA level Nitrite assay TNF-α BDNF	[26]
Lonchocarpine	Isolated from *Abrus precatorius*	Anti-inflammatory, anti-edematogenic, antibacterial, gastroprotective, and cytoprotective effects.	Male ICR mice (10–11 weeks), 50 mg/kg (i.p.)	5 mg/kg (i.p.)	Biochemical Estimation IL-6 IL-10 Nitrite assay TNF-α ROS level Western blot RT-PCR Immunohistochemistry Transient transfection and luciferase assay Co-immunoprecipitation assay	[27]
Trans-astaxanthin	Purchased from Sigma-Aldrich, USA	Anti-inflammatory and antioxidative activities.	Male ICR mice (4–6 weeks, 20–22 g); 20, 40, and 80 mg/kg (p.o.)	0.83 mg/kg (i.p.)	Behavioural Tail suspension test Forced swimming test Locomotor activity Biochemical Estimation IL-1β IL-6 iNOS, nNOS, eNOS level COX-2 level NF-κB p65 level Nitrite assay TNF-α RT-PCR	[28]
Proanthocyanidin (Found in algae, yeast, salmon, trout, krill, shrimpm and crayfish)	Purchased from Tianjin Jianfeng Natural Product R & D Co., Ltd. (Tianjin, China)	Anti-inflammatory and antioxidative activities.	Six-week-old male ICR mice (20–22 g), 80 mg/kg (p.o.)	0.83 mg/kg (i.p.)	Behavioural Forced swimming test Locomotor activity Marble-burying test Elevated plus maze test Biochemical Estimation IL-1β IL-6 iNOS level COX-2 level NF-κB p65 level TNF-α RT-PCR	[29]
Ginsenoside Rg3 (Found in *Panax ginseng*)	Purchased from the College of Chemistry, Jilin University 83 (Changchun, China).	Antioxidative, anti-inflammatory, and immunomodulatory effects.	Male ICR mice (8 weeks old), 20 and 40 mg/kg intragastric administration	0.83 mg/kg (i.p.)	Behavioural Forced swimming test Tail suspension test Open field test Biochemical Estimation IL-6 IL-1β IDO mRNA NF-κB level TNF-α RT-PCR Western blot	[30]
Paeoniflorin (Found in Paeonia, *Paeonia tenuifolia*)	Provided by Wako Pure Chemical Industries, Ltd. (Osaka, Japan)	Anti-inflammatory, anti-allergic, immunoregulatory, analgesic, neuromuscular blocking, cognition enhancement, and steroid protein-binding inhibition.	Female Sprague Dawley rats; 2.5, 5, and 10 mg/kg (i.p.) at a dose of 1.5 mL/kg	1.5 mL/kg (i.p.)	Biochemical Estimation Triglyceride (TG) Total cholesterol (TC) Total lipid (TL) High-density lipoproteins (HDLs) Malondialdehyde (MDA)	[31]
Hesperidin (Commonly found in citrus fruits)	Procured from Sigma, Aldrich USA	Anti-inflammatory, anti-apoptotic, and antioxidant activities.	Male Balb/c mice (8–10 weeks), 100 mg/kg (p.o.)	0.83 mg/kg (i.p.)	Behavioural Elevated plus Maze Light–dark box test Open field test Sucrose preference test Tail suspension test Forced swimming test Biochemical Estimation IL-1β IL-10 MDA level Nitrite level GSH level SOD and CAT activity Total protein CORT TNF-α BDNF RTPCR Western Blot	[32]
Carvacrol (Found in *Origanum vulgare, Thymus vulgaris,* and *Lepidium flavum*)	Purchased from Sigma-Aldrich Company (Sigma- Aldrich Co., St. Louis, MO, USA)	Antioxidative, anti-inflammatory, and anti-apoptotic effects.	Male Sprague–Dawley (SD) rat, (25, 50, and 100 mg/kg)	2 μL/1 min (total 5 min) intracerebroventricularly	Behavioural Object recognition task Morris water maze test Open field test Biochemical Estimation IL-1β Il-6 TNF-α COX-2 level NF-κB level iNOS level TLR4 level BDNF RTPCR	[33]
Quercetin (Found mostly in onions, grapes, berries, cherries, broccoli, and citrus fruits)	Purchased from Sigma-Aldrich Co., Steinheim, Switzerland	Anti-inflammatory, antiproliferative, and anti-atherosclerotic effects.	Female BALB/c inbred mice (7 weeks old), 0.06 or 0.15 μmol/mouse	8 and 16 mg/kg BW (i.p.)	Biochemical Estimation IL-6 IL-1β IL-17 IL-10 TNF-α	[34]
Isovitexin	Purchased from Sigma- Aldrich, St. Louis, MO, USA	Anti-inflammatory, antioxidant, and anxiolytic activities.	Male C57BL/6 mice (8–10 weeks and 20–25 g), 10 mg/kg (i.p.)	0.33 mg/kg (i.p.)	Behavioural Open field test Sickness behaviour Biochemical Estimation IL-6 RTPCR IL-1β IL-17 IL-10 TNF-α COX-2 iNOS Western blot	[35]
Solidagenone	*Solidago chilensis* Meyen	Anti-inflammatory, hypoglycaemic, analgesic, and hypolipidemic activities.	Male Swiss mice (25–35 g); 1, 10, or 100 mg/kg (p.o)	600 μg/kg (i.p.)	Behavioural Open field test Sickness behaviour Tail suspension test Biochemical Estimation MPO IL-6 TNF-α GSH level LOOH level SOD activity CAT activity GST activity	[36]
Diallyl disulfide	Purchased from Sigma	Anti-inflammatory and antioxidant activities.	Male C57BL6/J mice (6–8 weeks), 40 or 80 mg/kg (i.p.)	100 μg/kg (i.p.)	Behavioural Tail suspension test Forced swim test Open field test Biochemical Estimation IL-6 IL-1β TNF-α Nitric oxide level GSH level MDA level	[35]
Caffeic acid	Purchased from Sigma-Aldrich Co., LLC (St. Louis, MO, USA)	Antioxidant, antitumour, antinociceptive, antidementiam, and anti-inflammatory activities.	Male Swiss albino mice (8–10 weeks, 20–30 g), 30 mg/kg (p.o.)	1.5 mg/kg (i.p.)	Behavioural Open field test Forced swim test Tail suspension test Biochemical Estimation IL-6 TNF-α GSH level MDA level	[37]
Rosmarinic acid	Obtained from Sigma Aldrich Co. (St. Louis, MO, USA)	Anti-inflammatory, hepatoprotection, and renoprotection activities.	Adult Swiss albino mice, 0.5 mg/kg and 1 mg/kg (i.p.)	0.25 mg/kg (i.p.)	Behavioural Morris water maze Y maze Tail suspension test Biochemical Estimation SOD IL-6 TNF-α Caspase-3 C-Jun GSH level MDA level AChE activity TBARS assay	[38]
Parthenolide	Purchased from Sigma Chemicals, Deisenhofen, Germany	Anti-inflammatory and immunomodulatory effects.	Male Wistar rat, 1 mg/kg (i.p.)	100 μg/kg (i.p.)	Behavioural Morris water maze Y maze Tail suspension test Biochemical Estimation IL-6 TNF-α COX-2 level NF-κB/NF-IL6 pathway RTPCR PGC1a Trib1	[39]
Quercetin	Purchased from Sigma, St. Louis, MO, USA	Anti-inflammatory, antioxidant, antiallergic, antiapoptotic, nephro-, gastro-, angio-, cardio-, and chondroprotective properties.	Wistar albino rats, 2 and 25 mg/kg, (i.p.)	LPS, 1 mg/kg (i.p.)	Behavioural Elevated plus maze Open field test Biochemical Estimation IL-6 IL-1β TNF-α TBARS GSH	[40]
Embelin	*Embelia ribes* Burm	Anti-inflammatory, neuroprotective, anxiolytic, antitumour, analgesic, and anticonvulsant activities.	Adult male Swiss albino mice, 10 and 20 mg/kg (p.o.)	400 μg/kg (i.p.)	Behavioural Open field test Plus maze Light–dark box Forced swim test Social behaviour assessment Sucrose preference test Biochemical Estimation GSH level MDA level	[11]
25-methoxyhispidol	*Poncirus trifoliate*	Antibacterial, anti-inflammatory, and anticancer activities.	Male albino mice (3–4 weeks of age); 1, 5, and 10 mg/kg (i.p.)	0.83 mg/kg (i.p.)	Behavioural Elevated plus maze test Forced swim test Light dark box test Tail suspension test Open field test Biochemical Estimation IL-6 IL-1β TNF-α GSH level GST level ALT AST	[41]
Curcumin	Purchased from Sigma-Aldrich, Milan, Italy	Anti-inflammatory, antitumour, antioxidative, anti-amyloidogenic, metal-chelating, and cardiovascular protective effects.	3 month old male C57BL/6 mice, 50 mg/kg (p.o.)	5 mg/kg (i.p.)	Behavioural Open field test Novel object recognition test Biochemical Estimation IL-6 IL-1β TNF-α NLRP3 inflammasome COX-2 RTPCR	[42]
Liquiritigenin	Purchased from National Institutes for Food and Drug Control (Beijing, China)	Anti-inflammatory and neuroprotective activities.	ICR mice, 7.5 and 15 mg/kg intragastric	0.5 mg/kg (s.c.)	Behavioural Tail suspension test Forced swimming test Biochemical Estimation IL-6 TNF-α BDNF B(TrkB) Western blot RTPCR	[43]
Honokiol	Purchased from Sigma-Aldrich, St. Louis, MO, USA	Antioxidant, anti-inflammatory, anxiolytic, antidepressant, and neuroprotective activities.	Adult male Swiss albino mice, (22–30 g), 2.5 and5 mg/kg (i.p.)	0.83 mg/kg (i.p.)	Behavioural Tail suspension test Forced swim test Biochemical Estimation IL-6 IL-1β TNF-α BDNF CORT level TBARS level Nitrite level	[44]
Honokiol	Purchased from Sigma-Aldrich, St. Louis, MO, USA	Antiarrhythmic, anti-inflammatory, antithrombocytic, anti-angiogenesis, antitumour, anxiolytic, and antioxidative activities.	Adult male Swiss albino mice (22–30 g), 2.5 and 5 mg/kg (i.p.)	0.83 mg/kg (i.p.)	Behavioural Elevated plus maze test Open field test Biochemical Estimation IL-6 IL-10 IL-1β TNF-α BDNF AST level ALT level TBARS level GSH level	[24]
Paeonol	Provided by the National Institutes for Food and Drug Control (Beijing, China)	Anti-inflammatory, antioxidant, antiatherosclerosis, antidiabetic, antimutagenic agent, and antineuroinflammatory activities.	Male ICR mice, 10 and 20 mg/kg (i.p.)	0.5 mg/kg (i.p.)	Behavioural Forced swimming test Open field test Tail suspension test Biochemical Estimation 5-HT level NE level IL-6 TNF-α Western blot BDNF and NF-κB TrkB	[45]
Resveratrol	Purchased from Sigma-Aldrich (St. Louis, MO, USA)	Anti-inflammatory, antioxidant, and anti-anxiety activities.	Adult male C57BL/6J mice, 50 mg/kg (i.p.)	1 mg/kg (i.p.)	Behavioural Elevated plus maze test Open field test Morris water maze Biochemical Estimation IL-6 IL-1β IL-2 COX-2 iNOS NF-κB Western blot qRT-PCR	[46]
Ursolic acid	Purchased from Sigma-Aldrich (St. Louis, MO, USA)	Antioxidant, antitumour, and anti-inflammatory activities.	Male C57BL/6 mice, 10 mg/kg (p.o.)	10 or 20 mg/kg (i.p.)	Behavioural Step through passive avoidance test Open field test Morris water maze Biochemical Estimation IL-6 IL-1β COX-2 iNOS TNF-α NF-κB MAPK pathway *Akt* pathway	[47]
Curcumin	Purchased from Sigma–Aldrich	Anti-inflammatory, antioxidant, anticarcinogenic, and neuroprotective activities.	Adult Kun-Ming mice (male), 50 mg/kg (i.p.)	0.83 mg/kg (i.p.)	Behavioural Forced swimming test Tail suspension test Sucrose preference test Locomotor activity Biochemical Estimation IL-1β TNF-α COX-2 iNOS NF-κB Western blot RT-PCR NF-κB	[48]
Diallyl disulfide	Purchased from Sigma	Antimicrobial and anti-inflammatory activities.	Male C57BL6/J mice (6–8 weeks), 40 or 80 mg/kg (i.p.)	100 μg/kg (i.p.)	Behavioural Open field test Tail suspension test Forced swim test Biochemical Estimation IL-1β TNF-α Nitric oxide (NO) levels MDA level GSH level	[49]
Macranthol	*Illicium dunnianum* Tutch	Neuroprotective activities.	Male ICR mice, 20 mg/kg (p.o.)	0.83 mg/kg (i.p.)	Behavioural Sucrose preference test Forced swimming test Biochemical Estimation IL-1β IL-6 TNF-α qPCR iba1	[50]
Taraxasterol	Obtained from Chengdu Fenruisi BioTechnology Co. (Chengdu, China)	Antirheumatic, anti-inflammatory, and antimastopathy activities.	Male Kunming mice; 2.5, 5, and 10 mg/kg, intragastric	32 mg/kg (i.p.)	Behavioural Morris water maze test Passive avoidance Biochemical Estimation IL-6 IL-1β TNF-α IFN- γ MDA NO PGE2	[51]
Apelin	Purchased from Sigma-Aldrich Co. (St. Louis, MO, USA)	Antineuroinflammatory effects.	Male Wistar rats (200–220 g), 2 μg/kg (i.c.v.)	2 μg/kg (i.c.v.)	Behavioural Forced swimming test Sucrose preference test Passive avoidance Biochemical Estimation IL-1β TNF-α NF-κB p-IKKβ Western blot	[52]
Esculetin	Purchased from the National Institutes for Food and Drug Control (Beijing, China)	Antioxidant, anti-inflammatory, and hepatoprotective activities.	Male ICR mice (18–22 g), 20 and 40 mg/kg, intragastric administration	0.83 mg/kg (i.p.)	Behavioural Forced swimming test Tail suspension test Open field test Biochemical Estimation IL-6 IL-1β TNF-α COX-2 iNOS NF-κB BDNF p-TrkB Western blot RT-PCR NF-κB	[53]

**Table 2:** Mechanism of action(s) of HBACs against LPS-induced sickness behaviour in rodents.

HABC	Suppression of Oxidative Stress	Attenuation of Cytokine Levels	Suppression of Nitrosative Stress	COX-2 Inhibition	Suppression of Corticosterone	Restoration of BDNF Levels
Caffeic acid	Yes	Yes	-	-	-	-
Carvacrol	-	-	-	-	-	Yes
Curcumin	-	Yes	Yes	Yes	-	-
Diallyl disulfide	Yes	Yes	-	-	-	-
Ellagic Acid	Yes	-	-	-	-	-
Embelin	Yes	-	-	-	-	-
Esculetin	Yes	-	-	-	Yes	-
Gentiopicroside	-	Yes	-	-	-	-
Ginsenoside Rg3	-	Yes	-	-	-	-
Gomisin N	-	-	-	-	-	-
Gypenosides	-	-	-	-	-	Yes
Hesperidin	-	-	-	-	-	-
Honokiol	Yes	Yes	Yes	-	-	Yes
Isovitexin	-	-	-	-	-	-
Liquiritigenin	-	Yes	-	-	-	Yes
Lonchocarpine	-	Yes	-	-	-	-
Macranthol	-	Yes	-	-	-	-
Mangiferin	Yes	-	-	-	-	Yes
Methyl jasmonate	Yes	Yes	-	-	Yes	-
Paeoniflorin	Yes	-	-	-	-	-
Paeonol	-	Yes	-	-	-	Yes
Parthenolide	Yes	Yes	-	-	-	-
Curcumin + Piperine	Yes	-	Yes	-	-	-
Proanthocyanidin	-	Yes	-	Yes	-	-
Quercetin	Yes	Yes	-	-	-	-
Resveratrol	-	Yes	-	-	-	-
Rosmarinic acid	Yes	Yes	-	-	-	-
Selanylimidazopyridine	Yes	-	-	-	-	Yes
Solidagenone	Yes	-	-	-	-	-
Taraxasterol	-	Yes	-	-	-	-
Thymoquinone	Yes	Yes	-	-	-	-
Trans-astaxanthin	-	Yes	-	-	-	-
Ursolic acid	-	Yes	-	Yes	-	-
2, 3, 5, 4’-Tetrahydroxystilbene-2-O-β-D-glucoside	Yes	-	Yes	-	-	-
25-Methoxy hispidol	Yes	Yes	-	-	-	-

## References

[1] Alshehri S., Imam S.S. (2021). Rosinidin Attenuates Lipopolysaccharide-Induced Memory Impairment in Rats: Possible Mechanisms of Action Include Antioxidant and Anti-Inflammatory Effects. Biomolecules.

[2] Cunningham C., Maclullich A.M. (2013). At the extreme end of the psychoneuroimmunological spectrum: Delirium as a maladaptive sickness behaviour response. Brain Behav. Immun..

[3] Maes M., Berk M., Goehler L., Song C., Anderson G., Gałecki P., Leonard B. (2012). Depression and sickness behavior are Janus-faced responses to shared inflammatory pathways. BMC Med..

[4] Wilsterman K., Alonge M.M., Ernst D.K., Limber C., Treidel L.A., Bentley G.E. (2020). Flexibility in an emergency life-history stage: Acute food deprivation prevents sickness behaviour but not the immune response. Proc. R. Soc. B Biol. Sci..

[5] Prather A.A., Gellman M.D., Turner J.R. (2013). Sickness Behavior. Encyclopedia of Behavioral Medicine.

[6] Johnson R.W. (2002). The concept of sickness behavior: A brief chronological account of four key discoveries. Vet Immunol Immunopathol.

[7] McCusker R.H., Kelley K.W. (2013). Immune-neural connections: How the immune system’s response to infectious agents influences behavior. J. Exp. Biol..

[8] Turner M.D., Nedjai B., Hurst T., Pennington D.J. (2014). Cytokines and chemokines: At the crossroads of cell signalling and inflammatory disease. Biochim. Biophys. Acta (BBA) Mol. Cell Res..

[9] Eisenberger N.I., Moieni M., Inagaki T.K., Muscatell K.A., Irwin M.R. (2017). In Sickness and in Health: The Co-Regulation of Inflammation and Social Behavior. Neuropsychopharmacology.

[10] Dantzer R., BluthÉ R.-M., Castanon N., Kelley K.W., Konsman J.-P., Laye S., Lestage J., Parnet P., Ader R. (2007). CHAPTER 14—Cytokines, Sickness Behavior, and Depression. Psychoneuroimmunology.

[11] Shaikh A., Dhadde S.B., Durg S., Veerapur V.P., Badami S., Thippeswamy B.S., Patil J.S. (2016). Effect of Embelin Against Lipopolysaccharide-induced Sickness Behaviour in Mice. Phytother. Res. PTR.

[12] Maldonado R.F., Sá-Correia I., Valvano M.A. (2016). Lipopolysaccharide modification in Gram-negative bacteria during chronic infection. FEMS Microbiol. Rev..

[13] Lasselin J., Schedlowski M., Karshikoff B., Engler H., Lekander M., Konsman J.P. (2020). Comparison of bacterial lipopolysaccharide-induced sickness behavior in rodents and humans: Relevance for symptoms of anxiety and depression. Neurosci. Biobehav. Rev..

[14] Sadraie S., Kiasalari Z., Razavian M., Azimi S., Sedighnejad L., Afshin-Majd S., Baluchnejadmojarad T., Roghani M. (2019). Berberine ameliorates lipopolysaccharide-induced learning and memory deficit in the rat: Insights into underlying molecular mechanisms. Metab. Brain Dis..

[15] Wang M., Li H., Wang Y., Hao Y., Huang Y., Wang X., Lu Y., Du Y., Fu F., Xin W. (2020). Anti-Rheumatic Properties of Gentiopicroside Are Associated with Suppression of ROS-NF-κB-NLRP3 Axis in Fibroblast-Like Synoviocytes and NF-κB Pathway in Adjuvant-Induced Arthritis. Front. Pharm..

[16] Shivasharan B.D., Nagakannan P., Thippeswamy B.S., Veerapur V.P. (2013). Protective Effect of Calendula officinalis L. Flowers Against Monosodium Glutamate Induced Oxidative Stress and Excitotoxic Brain Damage in Rats. Indian J. Clin. Biochem. IJCB.

[17] Adebesin A., Adeoluwa O.A., Eduviere A.T., Umukoro S. (2017). Methyl jasmonate attenuated lipopolysaccharide-induced depressive-like behaviour in mice. J. Psychiatr. Res..

[18] Araki R., Hiraki Y., Nishida S., Inatomi Y., Yabe T. (2016). Gomisin N ameliorates lipopolysaccharide-induced depressive-like behaviors by attenuating inflammation in the hypothalamic paraventricular nucleus and central nucleus of the amygdala in mice. J. Pharmacol. Sci..

[19] Bargi R., Asgharzadeh F., Beheshti F., Hosseini M., Sadeghnia H.R., Khazaei M. (2017). The effects of thymoquinone on hippocampal cytokine level, brain oxidative stress status and memory deficits induced by lipopolysaccharide in rats. Cytokine.

[20] Chen Z., Huang C., He H., Ding W. (2017). 2, 3, 5, 4′-Tetrahydroxystilbene-2-O-β-D-glucoside prevention of lipopolysaccharide-induced depressive-like behaviors in mice involves neuroinflammation and oxido-nitrosative stress inhibition. Behav. Pharmacol..

[21] Deng Y.-t., Zhao M.-g., Xu T.-j., Jin H., Li X.-h. (2018). Gentiopicroside abrogates lipopolysaccharide-induced depressive-like behavior in mice through tryptophan-degrading pathway. Metab. Brain Dis..

[22] Domingues M., Casaril A.M., Birmann P.T., Lourenço D.d.A., Vieira B., Begnini K., Lenardão E.J., Collares T., Seixas F.K., Savegnago L. (2018). Selanylimidazopyridine Prevents Lipopolysaccharide-Induced Depressive-Like Behavior in Mice by Targeting Neurotrophins and Inflammatory/Oxidative Mediators. Front. Neurosci..

[23] Dornelles G.L., de Oliveira J.S., de Almeida E.J.R., Mello C.B.E., e Rodrigues B.R., da Silva C.B., Petry L.d.S., Pillat M.M., Palma T.V., de Andrade C.M. (2020). Ellagic Acid Inhibits Neuroinflammation and Cognitive Impairment Induced by Lipopolysaccharides. Neurochem. Res..

[24] Sulakhiya K., Kumar P., Gurjar S.S., Barua C.C., Hazarika N.K. (2015). Beneficial effect of honokiol on lipopolysaccharide induced anxiety-like behavior and liver damage in mice. Pharmacol. Biochem. Behav..

[25] Jangra A., Lukhi M.M., Sulakhiya K., Baruah C.C., Lahkar M. (2014). Protective effect of mangiferin against lipopolysaccharide-induced depressive and anxiety-like behaviour in mice. Eur. J. Pharmacol..

[26] Jangra A., Kwatra M., Singh T., Pant R., Kushwah P., Sharma Y., Saroha B., Datusalia A.K., Bezbaruah B.K. (2016). Piperine Augments the Protective Effect of Curcumin Against Lipopolysaccharide-Induced Neurobehavioral and Neurochemical Deficits in Mice. Inflammation.

[27] Jeong Y.-H., Park J.-S., Kim D.-H., Kang J.L., Kim H.-S. (2017). Anti-inflammatory mechanism of lonchocarpine in LPS- or poly(I:C)-induced neuroinflammation. Pharmacol. Res..

[28] Jiang X., Chen L., Shen L., Chen Z., Xu L., Zhang J., Yu X. (2016). Trans-astaxanthin attenuates lipopolysaccharide-induced neuroinflammation and depressive-like behavior in mice. Brain Res..

[29] Jiang X., Liu J., Lin Q., Mao K., Tian F., Jing C., Wang C., Ding L., Pang C. (2017). Proanthocyanidin prevents lipopolysaccharide-induced depressive-like behavior in mice via neuroinflammatory pathway. Brain Res. Bull..

[30] Kang A., Xie T., Zhu D., Shan J., Di L., Zheng X. (2017). Suppressive Effect of Ginsenoside Rg3 against Lipopolysaccharide-Induced Depression-Like Behavior and Neuroinflammation in Mice. J. Agric. Food Chem..

[31] Kim I.D., Ha B.J. (2010). The effects of paeoniflorin on LPS-induced liver inflammatory reactions. Arch. Pharmacal Res..

[32] Kwatra M., Ahmed S., Gawali B., Panda S.R., Naidu V.G.M. (2020). Hesperidin alleviates chronic restraint stress and lipopolysaccharide-induced Hippocampus and Frontal cortex damage in mice: Role of TLR4/NF-κB, p38 MAPK/JNK, Nrf2/ARE signaling. Neurochem. Int..

[33] Lee B., Yeom M., Shim I., Lee H., Hahm D.-h. (2019). Inhibitory effect of carvacrol on lipopolysaccharide-induced memory impairment in rats. KJPP.

[34] Liao Y.R., Lin J.Y. (2015). Quercetin intraperitoneal administration ameliorates lipopolysaccharide-induced systemic inflammation in mice. Life Sci..

[35] Liu B., Huang B., Hu G., He D., Li Y., Ran X., Du J., Fu S., Liu D. (2019). Isovitexin-Mediated Regulation of Microglial Polarization in Lipopolysaccharide-Induced Neuroinflammation via Activation of the CaMKKβ/AMPK-PGC-1α Signaling Axis. Front. Immunol..

[36] Locateli G., de Oliveira Alves B., Miorando D., Ernetti J., Alievi K., Zilli G.A.L., Serpa P.Z., Vecchia C.A.D., Mota da Silva L., Müller L.G. (2020). Antidepressant-like effects of solidagenone on mice with bacterial lipopolysaccharide (LPS)-induced depression. Behav. Brain Res..

[37] Basu Mallik S., Mudgal J., Nampoothiri M., Hall S., Dukie S.A., Grant G., Rao C.M., Arora D. (2016). Caffeic acid attenuates lipopolysaccharide-induced sickness behaviour and neuroinflammation in mice. Neurosci. Lett..

[38] Thingore C., Kshirsagar V., Juvekar A. (2021). Amelioration of oxidative stress and neuroinflammation in lipopolysaccharide-induced memory impairment using Rosmarinic acid in mice. Metab. Brain Dis..

[39] Rummel C., Gerstberger R., Roth J., Hübschle T. (2011). Parthenolide attenuates LPS-induced fever, circulating cytokines and markers of brain inflammation in rats. Cytokine.

[40] Sah S.P., Tirkey N., Kuhad A., Chopra K. (2011). Effect of quercetin on lipopolysaccharide induced-sickness behavior and oxidative stress in rats. Indian J. Pharmacol..

[41] Shal B., Khan A., Naveed M., Ali H., Seo E.K., Choi H., Khan S. (2020). Neuroprotective effect of 25-Methoxyhispidol A against CCl(4)-induced behavioral alterations by targeting VEGF/BDNF and caspase-3 in mice. Life Sci..

[42] Sorrenti V., Contarini G., Sut S., Dall’Acqua S., Confortin F., Pagetta A., Giusti P., Zusso M. (2018). Curcumin Prevents Acute Neuroinflammation and Long-Term Memory Impairment Induced by Systemic Lipopolysaccharide in Mice. Front. Pharm..

[43] Su Q., Tao W., Huang H., Du Y., Chu X., Chen G. (2016). Protective effect of liquiritigenin on depressive-like behavior in mice after lipopolysaccharide administration. Psychiatry Res..

[44] Sulakhiya K., Kumar P., Jangra A., Dwivedi S., Hazarika N.K., Baruah C.C., Lahkar M. (2014). Honokiol abrogates lipopolysaccharide-induced depressive like behavior by impeding neuroinflammation and oxido-nitrosative stress in mice. Eur. J. Pharmacol..

[45] Tao W., Wang H., Su Q., Chen Y., Xue W., Xia B., Duan J., Chen G. (2016). Paeonol attenuates lipopolysaccharide-induced depressive-like behavior in mice. Psychiatry Res..

[46] Tian Q., Fan X., Ma J., Han Y., Li D., Jiang S., Zhang F., Guang H., Shan X., Chen R. (2020). Resveratrol ameliorates lipopolysaccharide-induced anxiety-like behavior by attenuating YAP-mediated neuro-inflammation and promoting hippocampal autophagy in mice. Toxicol. Appl. Pharmacol..

[47] Wang Y.-J., Lu J., Wu D.-m., Zheng Z.-h., Zheng Y.-L., Wang X.-h., Ruan J., Sun X., Shan Q., Zhang Z.-f. (2011). Ursolic acid attenuates lipopolysaccharide-induced cognitive deficits in mouse brain through suppressing p38/NF-κB mediated inflammatory pathways. Neurobiol. Learn. Mem..

[48] Wang Z., Zhang Q., Yuan L., Wang S., Liu L., Yang X., Li G., Liu D. (2014). The effects of curcumin on depressive-like behavior in mice after lipopolysaccharide administration. Behav. Brain Res..

[49] Wei X., Ma Y., Li F., He H., Huang H., Huang C., Chen Z., Chen D., Chen J., Yuan X. (2021). Acute Diallyl Disulfide Administration Prevents and Reveres Lipopolysaccharide-Induced Depression-Like Behaviors in Mice via Regulating Neuroinflammation and Oxido-Nitrosative Stress. Inflammation.

[50] Weng L., Dong S., Wang S., Yi L., Geng D. (2019). Macranthol attenuates lipopolysaccharide-induced depressive-like behaviors by inhibiting neuroinflammation in prefrontal cortex. Physiol. Behav..

[51] Zhang X., Xiong H., Li H., Cheng Y. (2014). Protective effect of taraxasterol against LPS-induced endotoxic shock by modulating inflammatory responses in mice. Immunopharmacol. Immunotoxicol..

[52] Zhang Z.-x., Li E., Yan J.-p., Fu W., Shen P., Tian S.-W., You Y. (2019). Apelin attenuates depressive-like behavior and neuroinflammation in rats co-treated with chronic stress and lipopolysaccharide. Neuropeptides.

[53] Zhu L., Nang C., Luo F., Pan H., Zhang K., Liu J., Zhou R., Gao J., Chang X., He H. (2016). Esculetin attenuates lipopolysaccharide (LPS)-induced neuroinflammatory processes and depressive-like behavior in mice. Physiol. Behav..

[54] Seo D.Y., Lee S.R., Heo J.-W., No M.-H., Rhee B.D., Ko K.S., Kwak H.-B., Han J. (2018). Ursolic acid in health and disease. Korean J. Physiol. Pharm..

[55] Wirngo F.E., Lambert M.N., Jeppesen P.B. (2016). The Physiological Effects of Dandelion (Taraxacum Officinale) in Type 2 Diabetes. Rev. Diabet. Stud..

[56] Hewlings S.J., Kalman D.S. (2017). Curcumin: A Review of Its Effects on Human Health. Foods.

[57] Rinwa P., Kumar A. (2012). Piperine potentiates the protective effects of curcumin against chronic unpredictable stress-induced cognitive impairment and oxidative damage in mice. Brain Res..

[58] Singh S., Jamwal S., Kumar P. (2015). Piperine Enhances the Protective Effect of Curcumin Against 3-NP Induced Neurotoxicity: Possible Neurotransmitters Modulation Mechanism. Neurochem. Res..

[59] Woodbury A., Yu S.P., Wei L., Garcia P. (2013). Neuro-Modulating Effects of Honokiol: A Review. Front. Neurol..

[60] Imran M., Arshad M.S., Butt M.S., Kwon J.-H., Arshad M.U., Sultan M.T. (2017). Mangiferin: A natural miracle bioactive compound against lifestyle related disorders. Lipids Health Dis..

[61] Liang C., Ju W., Pei S., Tang Y., Xiao Y. (2017). Pharmacological Activities and Synthesis of Esculetin and Its Derivatives: A Mini-Review. Molecules.

[62] Sulakhiya K., Keshavlal G.P., Bezbaruah B.B., Dwivedi S., Gurjar S.S., Munde N., Jangra A., Lahkar M., Gogoi R. (2016). Lipopolysaccharide induced anxiety- and depressive-like behaviour in mice are prevented by chronic pre-treatment of esculetin. Neurosci. Lett..

[63] Espíndola K.M.M., Ferreira R.G., Narvaez L.E.M., Silva Rosario A.C.R., da Silva A.H.M., Silva A.G.B., Vieira A.P.O., Monteiro M.C. (2019). Chemical and Pharmacological Aspects of Caffeic Acid and Its Activity in Hepatocarcinoma. Front. Oncol..

[64] Dhadde S.B., Nagakannan P., Roopesh M., Anand Kumar S.R., Thippeswamy B.S., Veerapur V.P., Badami S. (2016). Effect of embelin against 3-nitropropionic acid-induced Huntington’s disease in rats. Biomed. Pharmacother..

[65] Thippeswamy B.S., Nagakannan P., Shivasharan B.D., Mahendran S., Veerapur V.P., Badami S. (2011). Protective effect of embelin from Embelia ribes Burm. against transient global ischemia-induced brain damage in rats. Neurotox. Res..

[66] Szopa A., Ekiert R., Ekiert H. (2017). Current knowledge of Schisandra chinensis (Turcz.) Baill. (Chinese magnolia vine) as a medicinal plant species: A review on the bioactive components, pharmacological properties, analytical and biotechnological studies. Phytochem. Rev..

[67] Ramalingam M., Kim H., Lee Y., Lee Y.-I. (2018). Phytochemical and Pharmacological Role of Liquiritigenin and Isoliquiritigenin From Radix Glycyrrhizae in Human Health and Disease Models. Front. Aging Neurosci..

[68] Himaya S.W.A., Ryu B., Qian Z.-J., Kim S.-K. (2012). Paeonol from Hippocampus kuda Bleeler suppressed the neuro-inflammatory responses in vitro via NF-κB and MAPK signaling pathways. Toxicol. Vitr..

[69] Jiang X., Zhu K., Xu Q., Wang G., Zhang J., Cao R., Ye J., Yu X. (2017). The antidepressant-like effect of trans-astaxanthin involves the serotonergic system. Oncotarget.

[70] Xu S., Liu J., Shi J., Wang Z., Ji L. (2017). 2,3,4′,5-tetrahydroxystilbene-2-O-β-D-glucoside exacerbates acetaminophen-induced hepatotoxicity by inducing hepatic expression of CYP2E1, CYP3A4 and CYP1A2. Sci. Rep..

[71] Kim J.-H. (2018). Pharmacological and medical applications of Panax ginseng and ginsenosides: A review for use in cardiovascular diseases. J. Ginseng Res..

[72] Jeong Y.-H., Park J.-S., Kim D.-H., Kim H.-S. (2016). Lonchocarpine Increases Nrf2/ARE-Mediated Antioxidant Enzyme Expression by Modulating AMPK and MAPK Signaling in Brain Astrocytes. Biomol. Ther..

[73] Arun M., Satish S., Anima P. (2016). Phytopharmacological Profile of Jasminum grandiflorum Linn. (Oleaceae). Chin. J. Integr. Med..

[74] Rauf A., Imran M., Abu-Izneid T., Iahtisham Ul H., Patel S., Pan X., Naz S., Sanches Silva A., Saeed F., Rasul Suleria H.A. (2019). Proanthocyanidins: A comprehensive review. Biomed. Pharmacother..

[75] Yang L., Xian D., Xiong X., Lai R., Song J., Zhong J. (2018). Proanthocyanidins against Oxidative Stress: From Molecular Mechanisms to Clinical Applications. BioMed. Res. Int..

[76] Domingues M., Casaril A.M., Smaniotto T.Â., Birmann P.T., Lourenço D.d.A., Bampi S.R., Vieira B., Lenardão E.J., Savegnago L. (2022). Selanylimidazopyridine abolishes inflammation- and stress-induced depressive-like behaviors by modulating the oxido-nitrosative system. Eur. J. Pharmacol..

[77] Chung H.J., Park E.J., Pyee Y., Hua Xu G., Lee S.H., Kim Y.S., Lee S.K. (2011). 25-Methoxyhispidol A, a novel triterpenoid of Poncirus trifoliata, inhibits cell growth via the modulation of EGFR/c-Src signaling pathway in MDA-MB-231 human breast cancer cells. Food Chem. Toxicol. Int. J. Publ. Br. Ind. Biol. Res. Assoc..

[78] Sy L.-K., Saunders R.M.K., Brown G.D. (1997). Phytochemistry of Illicium dunnianum and the systematic position of the illiciaceae. Phytochemistry.

[79] Li J., Geng D., Xu J., Weng L.J., Liu Q., Yi L.T. (2013). Antidepressant-like effect of macranthol isolated from Illicium dunnianum tutch in mice. Eur. J. Pharmacol..

[80] Luo L., Liu X.-L., Li J., Mu R.-H., Liu Q., Yi L.-T., Geng D. (2015). Macranthol promotes hippocampal neuronal proliferation in mice via BDNF-TrkB-PI3K/Akt signaling pathway. Eur. J. Pharmacol..

[81] Zanwar A.A., Badole S.L., Shende P.S., Hegde M.V., Bodhankar S.L., Watson R.R., Preedy V.R., Zibadi S. (2014). Chapter 76—Cardiovascular Effects of Hesperidin: A Flavanone Glycoside. Polyphenols in Human Health and Disease.

[82] Andrade S., Ramalho M.J., Pereira M.d.C., Loureiro J.A. (2018). Resveratrol Brain Delivery for Neurological Disorders Prevention and Treatment. Front. Pharm..

[83] Kuršvietienė L., Stanevičienė I., Mongirdienė A., Bernatonienė J. (2016). Multiplicity of effects and health benefits of resveratrol. Medicina.

[84] Vasconcelos J.F., Santos I.P., de Oliveira T.B., Kelly A.M., do Reis B.P.Z.C., Orge I.D., Meira C.S., Valverde S.S., Soares M.B.P. (2022). The protective effect of solidagenone from Solidago chilensis Meyen in a mouse model of airway inflammation. Basic Clin. Pharmacol. Toxicol..

[85] Bortoleti B.T.d.S., Gonçalves M.D., Tomiotto-Pellissier F., Contato V.M., Silva T.F., de Matos R.L.N., Detoni M.B., Rodrigues A.C.J., Carloto A.C., Lazarin D.B. (2021). Solidagenone acts on promastigotes of L. amazonensis by inducing apoptosis-like processes on intracellular amastigotes by IL-12p70/ROS/NO pathway activation. Phytomedicine.

[86] Bayan L., Koulivand P.H., Gorji A. (2014). Garlic: A review of potential therapeutic effects. Avicenna J. Phytomed..

[87] Lu J., He H., Huang C., Chen Z. (2019). Effect of Diallyl Disulfide on Lipopolysaccharide-Induced Depression-Like Behavior in Mice. https://www.researchsquare.com/article/rs-10369/v1.

[88] De Oliveira J.R., Camargo S.E.A., de Oliveira L.D. (2019). *Rosmarinus officinalis* L. (rosemary) as therapeutic and prophylactic agent. J. Biomed. Sci..

[89] Du W., Liang X., Wang S., Lee P., Zhang Y. (2020). The Underlying Mechanism of *Paeonia lactiflora* Pall. in Parkinson’s Disease Based on a Network Pharmacology Approach. Front. Pharm..

[90] Tizard I. (2008). Sickness behavior, its mechanisms and significance. Anim. Health Res. Rev..

[91] Pareek A., Suthar M., Rathore G.S., Bansal V. (2011). Feverfew (*Tanacetum parthenium* L.): A systematic review. Pharm. Rev.

[92] Goyal S.N., Prajapati C.P., Gore P.R., Patil C.R., Mahajan U.B., Sharma C., Talla S.P., Ojha S.K. (2017). Therapeutic Potential and Pharmaceutical Development of Thymoquinone: A Multitargeted Molecule of Natural Origin. Front. Pharm..

[93] Khader M., Eckl P.M. (2014). Thymoquinone: An emerging natural drug with a wide range of medical applications. Iran. J. Basic Med. Sci..

[94] Li Y., Lin W., Huang J., Xie Y., Ma W. (2016). Anti-cancer effects of Gynostemma pentaphyllum (Thunb.) Makino (Jiaogulan). Chin. Med..

[95] Shin K.S., Zhao T.T., Park K.H., Park H.J., Hwang B.Y., Lee C.K., Lee M.K. (2015). Gypenosides attenuate the development of L-DOPA-induced dyskinesia in 6-hydroxydopamine-lesioned rat model of Parkinson’s disease. BMC Neurosci..

[96] Lee B., Shim I., Lee H. (2018). Gypenosides Attenuate Lipopolysaccharide-Induced Neuroinflammation and Memory Impairment in Rats. Evid. Based Complement. Altern. Med..

[97] Zhang Y., Qi Z., Wang W., Wang L., Cao F., Zhao L., Fang X. (2021). Isovitexin Inhibits Ginkgolic Acids-Induced Inflammation Through Downregulating SHP2 Activation. Front. Pharm..

[98] Sharifi-Rad M., Varoni E.M., Iriti M., Martorell M., Setzer W.N., Del Mar Contreras M., Salehi B., Soltani-Nejad A., Rajabi S., Tajbakhsh M. (2018). Carvacrol and human health: A comprehensive review. Phytother. Res. PTR.

[99] Javed H., Meeran M.F.N., Jha N.K., Ojha S. (2021). Carvacrol, a Plant Metabolite Targeting Viral Protease (Mpro) and ACE2 in Host Cells Can Be a Possible Candidate for COVID-19. Front. Plant Sci..

[100] Baliga M.S., Shivashankara A.R., Venkatesh S., Bhat H.P., Palatty P.L., Bhandari G., Rao S., Watson R.R., Preedy V.R. (2019). Chapter 7—Phytochemicals in the Prevention of Ethanol-Induced Hepatotoxicity: A Revisit. Dietary Interventions in Liver Disease.

[101] Miranda M., Morici J.F., Zanoni M.B., Bekinschtein P. (2019). Brain-Derived Neurotrophic Factor: A Key Molecule for Memory in the Healthy and the Pathological Brain. Front. Cell. Neurosci..

[102] Bathina S., Das U.N. (2015). Brain-derived neurotrophic factor and its clinical implications. Arch. Med. Sci. AMS.

